# Physical activity and stress among college students at a large US university during the COVID‐19 pandemic

**DOI:** 10.1002/puh2.124

**Published:** 2023-09-30

**Authors:** Wendy DeYoung, Becca Schulte, Kaigang Li

**Affiliations:** ^1^ Department of Health and Exercise Science Colorado State University Fort Collins Colorado USA; ^2^ Colorado School of Public Health Fort Collins Colorado USA

**Keywords:** college students, COVID‐19 pandemic, perceived stress, physical activity, sitting time, trends

## Abstract

**Background:**

As the onset of the COVID‐19 pandemic and subsequent lockdown in March 2020, reports indicate reduced physical activity (PA), increased sitting time (ST), and higher stress in various groups. It remains uncertain if these patterns persist in college students. This study aimed to discover trends in vigorous PA (VPA), moderate PA (MPA), light PA (LPA), ST, and stress among college students from 2020 to early 2022, spanning 2 years.

**Methods:**

Using a repeated cross‐sectional design, this study involved three online surveys in Summer and Fall 2020, and Winter 2021, respectively. Participants recalled pre‐COVID‐19 information in the first two periods and reported current information during COVID in all three periods, outlining four time points: Before‐COVID and During‐COVID times 1, 2, and 3.

**Results:**

This study included 2163 students from a large Western university. All types of PA decreased from the start of the pandemic to late 2021; however, increases toward pre‐pandemic levels in MPA and LPA were found in early 2022. Also, activity shifted from VPA to MPA and LPA. Although ST and perceived stress (PS) surged in the pandemic's first year, they began decreasing toward pre‐COVID levels in early 2022.

**Conclusion:**

The study indicates a decline in PA during the peak of the pandemic, followed by a recent return to almost pre‐COVID levels. Additionally, elevated ST and PS in early pandemic have reduced by the third year. As society begins to live with a new COVID‐normal, there must be adaptations to maintain PA and promote mental well‐being.

## Graphical abstract

We examined physical activity, sitting time (ST), and perceived stress among college students over 2 years during the COVID. The findings indicate a decline in PA during the peak of the pandemic, followed by a recent return to almost pre‐COVID levels and a reduction of elevated ST and perceived stress.

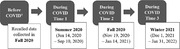

## INTRODUCTION

The World Health Organization (WHO) declared COVID‐19 a global pandemic in March 2020 [1, 2], and it soon became clear no place would be spared. Countries and regions around the world instated social distancing, masking, and lockdowns. Although the United States (US) never had a country‐wide lockdown, many states implemented “stay at home” orders or other forms of restrictions [3]. At that time, most thought that these measures would be temporary and life would soon return to normal. Two years later, there have been over 500 million global cases and over 6 million deaths, with versions of restrictions being revoked and re‐implemented throughout that time [4].

The pandemic has brought drastic changes in many aspects of human life. Lockdowns, restrictions, and quarantine impact people's work, education, financial situation, social lives, travel, and recreation [5, 6, 7, 8]. As a consequence, there were disruptions to individuals’ daily habits, behaviors, and overall well‐being. Two main areas of disruption were on physical activity (PA) and mental health. These were demonstrated by a plethora of studies looking at the impact of COVID‐19 on PA [9, 10, 11, 12]. Most of these studies were conducted during the initial stages of the pandemic of spring 2020, probably to take advantage of the prime opportunity for unique research. Fewer research studies have been published on longitudinal trends, specifically past 2020.

The majority of studies show a general trend of decreasing total amount of PA when comparing “during pandemic” to “pre‐pandemic” [13]. There also appears to be a trend of decreasing intensity [14]. It should be noted that while on average PA decreased, in most if not all studies, some percentage of the population increased their PA. Furthermore, the degree of change ranges largely and may not always be of a significant amount. On one hand, a French cross‐sectional study found on average an 8‐min decrease in moderate‐to‐vigorous PA (MVPA) per week during the lockdown compared to the week before the lockdown [15]. A difference of 8 min per week may not be large enough to see clinical changes in health or well‐being. However, another large‐scale study in the United Kingdom using data from smartphone app designed for activity tracking reported an average decrease of 124 minutes per week in overall physical activity [16]. Considering the Physical Activity Guidelines for Americans state adults should strive for at least 150 min of PA a week, a decrease this large could have drastic impacts on health and well‐being [17, 18].

When narrowing the scope to young adults, findings are similar. A US longitudinal study of emerging adults found that MVPA had decreased 1.2 h a week and total PA has decreased on average by 2.1 h a week [19]. A US study of university students found a sharp decline in the average number of steps a day dropping from an average 10,000 steps to 4600 steps per day [20]. Additionally, non‐sedentary activities dropped by 1.5 h per day. A study of high school and university students in China found a decrease in active transportation (0.3 h/week) and leisure time MVPA (0.1 h/week) [21].

Not surprisingly, mental health has worsened with the pandemic. The initial emergence of a novel virus with a rapid transmission, relatively high mortality rate, and the subsequent life‐altering consequences has exacerbated stress and anxiety among the general population [22]. There have been higher rates and dramatic increases of depressive and anxiety symptoms, increases in perceived stress (PS) and loneliness, and an overall decrease in mental well‐being in young adults and adults [20, 23, 24]. Research studies specific to college students have found a variety of health concerns, including a 61% increase in depression over the course of the 2020 spring semester compared to a 12% increase the year before [20]. Instances of alcohol use disorder and bulimia nervosa were notably higher during the pandemic than pre‐pandemic [25]. Moreover, a survey study at a Texas university found that 71% of students experienced heightened stress and anxiety due to the COVID‐19 pandemic [26].

Although at one point it may have been hoped that we would return to “normal,” it now seems apparent that we have entered a “new normal” where not only severe acute respiratory syndrome coronavirus 2 (SARS‐CoV‐2) will continue to circulate and mutate, but its lifestyle‐changing consequences are here to stay [27]. It is unclear at this point how PA and mental health are adapting.

As of the beginning of 2022, there is limited evidence on how PA trends have continued throughout the pandemic. Most of the initial research studies involved measured behavior specifically during “lockdown” periods. A couple of studies have investigated longer term trends. A French study found that the sharpest declines in PA happened initially during lockdown‐specific periods, suggesting that the dramatic changes may not be long lived and conditional upon closed gyms and recreational spaces and the novelty of the pandemic [15]. A US study with an adult population supplies some evidence of a “bounce back” or return to PA as the number of days exercised decreased from April to December 2020, but then increased by June 2021, though still lower than initial [28]. When looking specifically at university students, a longitudinal cohort study of students in the United Kingdom found decreased MVPA in October 2020 but does not provide further trends at this time [29]. On one hand, it would make sense for PA trends to go back to a pre‐pandemic state as the pandemic recedes. On the other hand, it would not be surprising that after the length of time that the disruption has occurred, the decreased PA trends have become the new “normal.” Regardless, currently a limited number of observations exist to specify the long‐term behavior change impacts. Similarly, limited evidence exists on whether these worsening mental health trends have continued.

In summary, it is still unclear from the literature whether these trends in PA and mental health have been sustained as the pandemic continues. The aim of the current study was to provide updated findings regarding these behavior and lifestyle trends among college students. Using a repeated cross‐sectional study design with pre‐COVID and multiple during‐COVID measurements of activity levels and stress levels among college students at a large Midwest university, this study provides key insights to how society is adapting to a “new normal.”

## METHODS

### Study design, setting, and population

**FIGURE 1:**
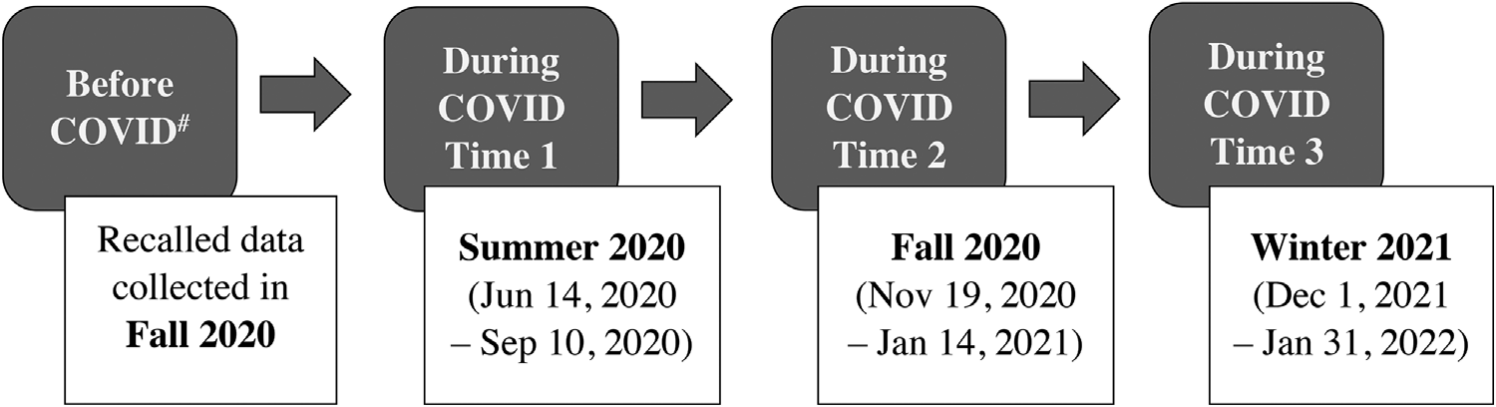
Data collection periods. *Note*: Four data points were used for the analysis: Before COVID (^#^data collected in Fall 2020 were used in the analysis because perceived stress data collected in Summer 2020 were not available), During COVID Time 1, During COVID Time 2, and During COVID Time 3.

This study was an online cross‐sectional survey conducted in the College of Health and Human Sciences at Colorado State University, a large Western university in the US. The survey was completed online through the web‐based platform Qualtrics. This survey was administrated during three time periods (Figure 1): Summer 2020 (June 14, 2020 to September 10, 2020), Fall 2020 (November 19, 2020 to January 14, 2021), and Winter 2021 (December 1, 2021 to January 31, 2022). For the first two periods, participants were asked to recall the information before COVID‐19 and report current information during COVID‐19; whereas for the third period, participants were asked to report the current information only. We retrieved the data collected from the three periods to indicate four time points: Before COVID (using the data collected in Fall 2020 because PS data were collected in this period only), During COVID Time 1 (Summer 2020), During COVID Time 2 (Fall 2020), and During COVID Time 3 (Winter 2021). The online survey took approximately 10 min to complete.

### Data gathering

An email invitation was extended to all undergraduate and graduate students at the said college, using their university email accounts. The study was open to all enrolled students, and those who provided consent for participation were included in the analysis. Three relatively equally spaced email reminders were sent during each survey period.

### Study variables

#### Demographic characteristics

Demographic data collected included age, sex (male vs. female), race/ethnicity (non‐White vs. White), and student category (first‐generation vs. non‐first‐generation).

#### Physical activity and sedentary behavior

Self‐reported PA and sitting time (ST) in the last 7 days were measured using the validated International Physical Activity Questionnaire (IPAQ) [30]. The information regarding vigorous PA (VPA), moderate PA (MPA), light PA (LPA), and ST before and during COVID‐19 was collected. Total minutes per week of VPA, MPA, LPA, and ST before and during COVID‐19 was collected and calculated. The data were processed based on the Guidelines for Data Processing and Analysis of the IPAQ [31]. Specifically, all cases were excluded if the total of all VPA, MPA, and LPA variables were greater than 960 min (16 h) based on the assumption that on average an individual of 8 h per day is spent sleeping. A new variable was created truncating MPA and VPA to 180 min each. This rule permits a maximum of 21 h of activity in a week to be reported for each category (3 h × 7 days).

#### Perceived stress

Self‐reported perceived stress (PS) in the last month was measured using the validated Perceived Stress Scale (PSS) [32]. Participants reported the degree to which situations in one's life have been unpredictable, uncontrollable and overloaded in the past month (e.g., ‘‘In the last month, how often have you felt nervous and “stressed”?) on a 5‐point Likert scale (0 = never, 1 = almost never, 2 = sometimes, 3 = fairly often, and 4 = very often). Scores for the four positively stated items (Items 4, 5, 7, 8) are reversed. Individual scores on the PSS can range from 0 to 40 with a higher score indicating higher PS.

### Data analysis

Data analysis was performed with IBM SPSS 26 [33] and SAS v9.4 (SAS Institute, Cary, NC). Descriptive statistics (i.e., mean, standard deviation, frequency, and proportion) were calculated to describe the demographic information and self‐reported PA, ST, and PS. General linear model was used to compare the data between four time points, including one time point before COVID and three time points during COVID‐19. A *p*‐value of ≤0.05 was deemed statistically significant, and the Bonferroni correction was used for multiple comparisons.

### Ethical considerations

The research project was reviewed and approved by the University Institutional Review Board (20‐10097H). Informed consent was agreed on as participants activated the survey with the link provided in the announcement.

## RESULTS

Table 1 describes the study population. A total of 2163 students (*N* = 648 in Summer 2020, *N* = 796 in Fall 2020, and *N* = 719 in Winter 2021) across the three time periods consented to participate in the study. The 796 participants in Fall 2020 were included in the analysis twice because the information they recalled for the Before COVID and the current information that they reported for During COVID Time 2 were used. Most participants were female, White, and non‐first‐generation students. Almost 50% of the participants were 22 years or older when taking the survey.

**TABLE 1:** Participant characteristics.

	*n* ^a^	%
Sex		
Male	478	21.25
Female	1742	77.46
Gender nonconforming/nonbinary	29	1.29
Race		
White	1779	79.03
Others	472	20.97
First generation student		
Yes	594	26.41
No	1655	73.59
Age		
18 Years or younger	308	13.66
19 Years	293	13.00
20 Years	270	11.98
21 Years	295	13.09
22 Years or older	1088	48.27

^a^ The *n* indicates instances involving individuals not distinct persons. Additionally, it is important to acknowledge that the total *n* for each variable might vary and not encompass the complete person‐times due to missing data.

**FIGURE 2:**
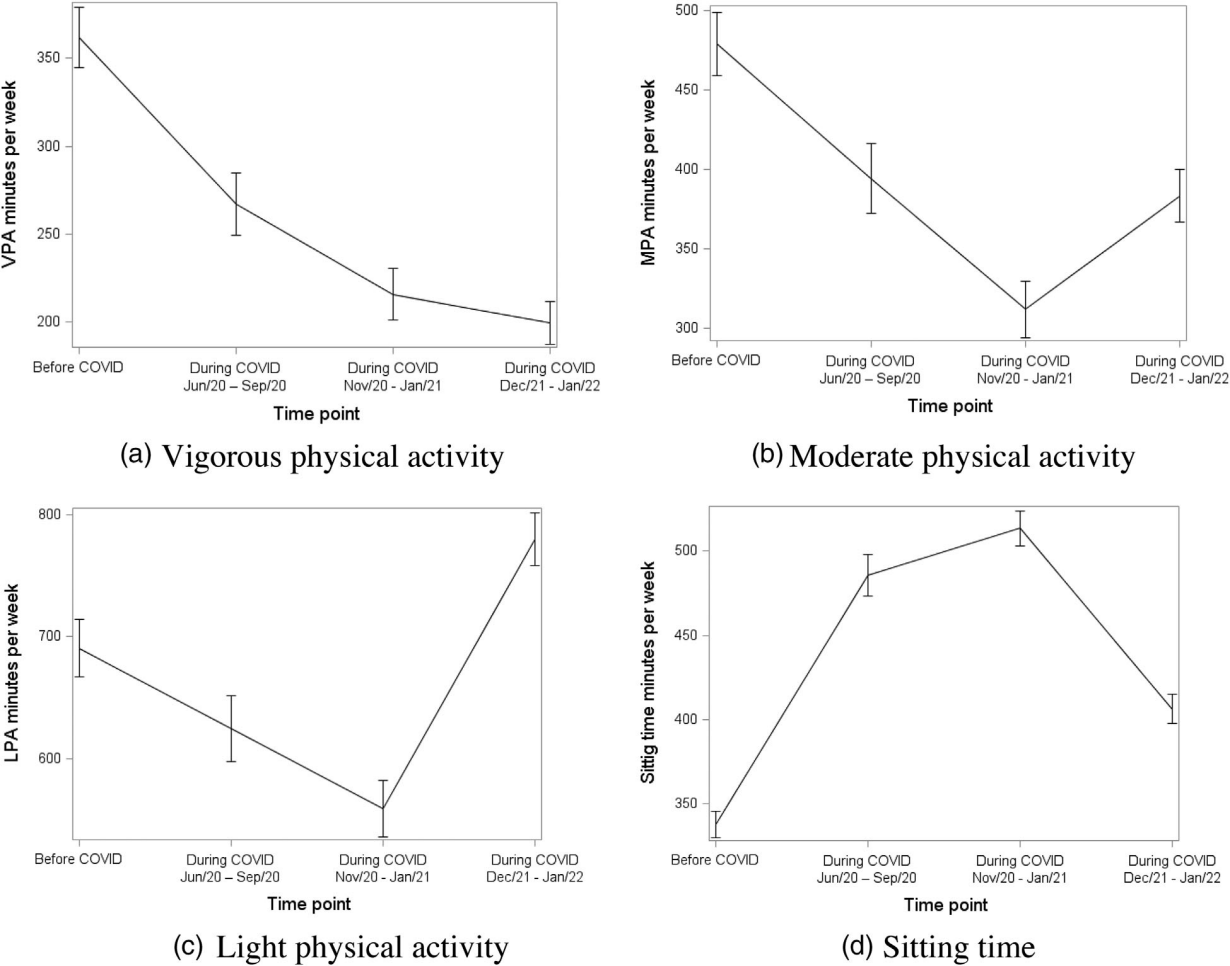
Trend in physical activity and sitting time among college students from before to three time points during COVID pandemic: (a) vigorous physical activity, (b) moderate physical activity, (c) light physical activity, and (d) sitting time.

**FIGURE 3:**
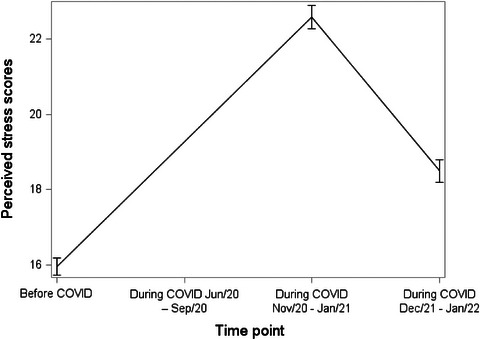
Trend in perceived stress among college students from before to three time points during COVID pandemic.

Table 2 shows the number of responses, mean, and standard deviation for each PA measure, ST, and PS at each time point. The table also indicates whether the difference compared to Before COVID is statistically significant. These trends in measures are visually summarized in Figures 2a–d and 3. For all measures, when compared to Before COVID, the difference was significant.

**TABLE 2:** Means of student vigorous physical activity (VPA), moderate physical activity (MPA), light physical activity (LPA), sitting time (ST), and perceived stress scores (PSS) over four time points.

	Before COVID^a^			During COVID Time 1			During COVID Time 2			During COVID Time 3		
	*N*	M	SD	*N*	M	SD	*N*	M	SD	*N*	M	SD
VPA	408	361.88	347.48	321	267.10^b^	320.21	414	215.80^b^	296.09	452	199.38^b^, ^c^	259.57
MPA	393	478.70	392.53	304	394.00^b^	385.86	400	311.51^b^, ^c^	358.56	449	383.08^b^, ^d^	350.69
LPA	384	690.39	461.47	295	624.61^b^	466.25	391	558.99^b^	455.51	405	779.59^b–d^	438.53
ST	418	337.57	158.59	328	485.72^b^	221.85	419	513.51^b^	211.67	469	406.41^b–d^	183.15
PSS^e^	482	15.95	5.09	–	–	–	482	22.59^b^	6.72	502	18.50^a^, ^c^	6.65

*Note*: Before COVID: before March 2020; During COVID Time 1: 6/14/2020 to 9/10/2020 (Summer 2020); During COVID Time 2: 11/19/2020 to 1/14/2021 (Fall 2020); During COVID Time 3: 12/1/2021 to 1/31/2022 (Winter 2021).

Abbreviation: M: mean; SD = standard deviation.

^a^ Before COVID Data were collected in Fall 2020 when participants were asked to recall their behaviors Before COVID.

^b^ Compared to Before COVID (*p* < 0.05).

^c^ Compared to During COVID Time 1 (*p* < 0.05).

^d^ Compared to During COVID Time 2 (*p* < 0.05).

^e^ Before COVID PSS Data were collected in Fall 2020 when participants were asked to recall their behaviors before COVID.

The average amount of VPA was highest Before COVID at 361.88 min (SD = 357.48) and subsequently decreased at During COVID Times 1, 2, and 3 to 268.1 (SD = 320.21), 215.8 (SD = 296.09), and 199.38 (SD = 259.57) min, respectively (Table 2, Figure 2a).

MPA showed a similar trend dropping from an average of 478.7 min Before COVID (SD = 392.53) to 311.51 (SD = 358.56) min at During COVID Time 2; however, the average minutes did increase at During COVID Time 3 to 383.08 min (SD = 350.69). Regardless, this average is still almost 100 min less than Before COVID (Table 2, Figure 2b).

LPA decreased from an average 690.39 min Before COVID to 558.99 min (SD = 455.51) at During COVID Time 2, but surprisingly at During COVID Time 3 had the highest average total minutes at 779.59 (SD = 438.53) (Table 2, Figure 2c).

Sitting time increased from Before COVID at an average of 337.57 min (SD = 158.59) to 485.72 min (SD = 221.85) at During COVID Time 1 and 513.51 min (SD = 211.67) 2. By During COVID Time 3, the average ST receded to 406.41 min (SD = 183.15). However, it was still higher than the initial average Before COVID (Table 2, Figure 2d).

PS had a sharp increase from an average score of 15.95 (SD = 5.09) Before COVID to 22.59 (SD = 6.72) at During COVID Time 2 but similarly returned closer to the baseline by During COVID Time 3 with an average score of 18.5 (SD = 6.65) (Table 2, Figure 3).

## DISCUSSION

This study evaluated long‐term changes in PA and PS in college students through a 19‐month period (June 2020 to Jan 2022) of the COVID‐19 pandemic using data collected from repeated cross‐sectional surveys at a large Western university. The results indicate that the pandemic brought about significant disruption in daily habits, resulting in decreased PA and increased PS at the early stage of the pandemic. At all‐time points during COVID, the mean MPA and VPA minutes were significantly lower than before COVID. This is consistent with the previous research showing an overall decrease, specifically at early parts in the pandemic [13]. Our study extended beyond most current research to indicate longer term PA trends. At later stages in the pandemic, we found evidence of an increase in PA returning to previous levels. MPA was higher at time point 3 than it had been at time points 1 and 2. Furthermore, LPA was higher than pre‐COVID levels.

Some studies have disaggregated data based on individual pre‐pandemic activity levels and shown that the change in activity depends on prior engagement. Three separate studies found that PA decrease was largest for those previously active individuals, whereas previously low/inactive individuals saw little/smaller change in their activity levels [16, 34, 35]. The relative total amount of PA of these participants was high (with averaging over 1000 min of PA a week), so if following worldwide trends, the large decrease in PA among these participants is not surprising.

It is not clear from these results or others as the precise reasoning behind an individual's reduction in PA. Nevertheless, some speculations can be made due to the upheaval the COVID‐19 pandemic brought. Originally, gyms and recreational facilities, including the student recreational facility, were closed, potentially disrupting many people's PA routines [10, 36, 37]. Even when gyms were reopening, some may have felt uncomfortable due to potentially exposing themselves or not wanting to work out while wearing a mask (as required at this university). This may be especially true after reports of outbreaks being linked to gyms [38]. Additionally, many people's daily routines changed due to working (or schooling) from home. Active commuting significantly increases total PA [39]. University students, typically using forms of active transportation to get around campus and social activities, likely greatly reduced their movement about campus with remote learning and reduced academic and group events. Other reasons contributing to decreased PA could be related to lack of motivation and/or self‐efficacy, lack of organized sports and clubs, and general life stress [10, 37].

There is some evidence that over time the makeup of PA intensity is adapting. Total activity may be “bouncing back” at lower intensities. VPA was at its lowest of the time points at time point 3, with an average of under 200 min per week. On the other hand, for moderate activity, the average minutes had increased from time period 2. The average minutes were still much lower than before COVID‐19. Furthermore, although LPA did initially decrease, at the latest time point, the average was *higher* than pre‐COVID. When adding VPA, MPA, and LPA together, the low point was at time point 2. Although the average total PA minutes from the recent survey is still 170 min less than before the onset of COVID‐19, the trend is increasing. These results suggest that students seem to be increasing the amount of PA back toward pre‐COVID levels, but at a lower intensity. This potential shift is supported with the previous early pandemic research. Flanagan et al. found that the intensity, in addition to total time of PA, decreased with the onset of the pandemic [14]. Additionally, some initial COVID studies found that light PA, such as walking, housework, or yard work increased [21, 40–42].

The trends for ST reciprocate that of PA, such that through time points 1 and 2, there was an increase, but by time point 3, ST appears to be waning. However, it remains higher than previous trends. One reason for the increase in ST may be due to remote learning. At time points 1 and 2, students were receiving instruction remotely [43, 44]. This means that there is no necessity in getting up and walking to class every day, but students could choose to remain in their dorm/at home to participate in class and studying [36]. During time point 3, many classes (though not all) had returned to a more normal in person scenario [45], lending itself to less time in one place.

For PS, participants felt a large increase in stress from before COVID‐19 to time 2. This is consistent with previous findings of poorer mental health and increased stress as a result of the pandemic [22, 26]. By time 3, some of this average heightened stress had subsided. At time 2, in late 2020, the big winter wave was upon the US. After what might have been a promising summer, it was becoming a reality that the COVID‐19 was not going anywhere. Although vaccinations were on the brink, slim to none of the participants in the survey would have been vaccinated at this point. This new wave may have brought about increased fear, anxiety, and stress to everyday lives. By time point 3, although another wave was emerging thanks to the omicron variant, many students were vaccinated (as required by the university) [46], and the stress of the enduring crisis may have subsided.

This study is one of the first of its kind to track long‐term impacts of the COVID‐19 pandemic on PA and PS. Although the literature is saturated with short‐term impacts of lockdown measures, long‐term trends have yet to be published. This study greatly contributes to the growing literature on COVID, PA, and mental health.

This study is not without its limitations. First, our data collection relied on self‐report surveys, which are subject to bias. Because we gathered information on current PA and stress levels at later time points, assessing “before COVID” conditions retroactively may introduce recall bias. Second, due to the absence of identifiers in the survey, we were unable to employ longitudinal analysis methods, even though many students participated in the study at various time points. Lastly, it is important to note that our study exclusively focused on from data from college students attending a single university, which may not be generalizable to the entire population.

The study highlights the significant impact of the COVID‐19 pandemic on college students' PA levels and mental well‐being and has important implication for public health research and practice. Despite the recent positive trends, there is a need to recognize the ongoing challenges associated with living in a post‐pandemic society. As the world adjusts to a new normal with COVID‐19, it is crucial to implement strategies to maintain PA levels and promote mental well‐being among college students. This could include providing accessible resources and support for maintaining active lifestyles and addressing mental health concerns. By adopting adaptive measures, educational institutions can help their students cope with the long‐term effects of the pandemic and prioritize their overall health and well‐being.

## CONCLUSIONS

The first year of the pandemic brought about decreased PA, increased ST, and increased stress in a sample of college students. After 19 months of the pandemic, total PA is nearing pre‐COVID levels though the makeup has shifted to more LPA and less VPA. In contrast, ST and PS decreased early in the third year. Regardless, considering the importance of PA and mental health among college students, public health officials and health educators should continue to promote PA and tracking methods and provide resources for addressing mental health in this population.

## AUTHOR CONTRIBUTIONS

*Conceptualization; data curation; investigation; methodology; project administration; resources; writing—review and editing*: Wendy DeYoung and Kaigang Li. *Data curation; formal analysis; writing—original draft; writing—review and editing*: Becca Schulte, Kaigang Li, and Wendy DeYoung.

## CONFLICT OF INTEREST STATEMENT

The authors have no conflicts of interest to disclose.

## FUNDING INFORMATION

No funding was received for this manuscript.

## ETHICS STATEMENT

The data presented here have not been published elsewhere and all research activities were approved by the Institutional Review Board at the Colorado State University.

## Data Availability

The data that support the findings of this study are available from the corresponding author upon reasonable request.
